# Effects of fixed cutoff filtering on dark- and light-adapted ERG components and the application of variable cutoff filter

**DOI:** 10.1007/s10633-021-09853-9

**Published:** 2021-09-24

**Authors:** Min Gao, Mirella Telles Salgueiro Barboni, Dora Fix Ventura, Balázs Vince Nagy

**Affiliations:** 1 Department of Mechatronics, Optics and Mechanical Engineering Informatics, Budapest University of Technology and Economics, Budapest, Hungary; 2 Department of Ophthalmology, Semmelweis University, Budapest, Hungary; 3 Department of Experimental Psychology, University of Sao Paulo, Sao Paulo, Brazil

**Keywords:** Retina, Electroretinogram, Oscillatory potentials, ISCEV standard, Spectral analysis, Cutoff frequency, Variable filter

## Abstract

**Purpose:**

Human oscillatory potentials (OPs) are derived from dark-adapted (DA) electroretinograms (ERGs) with fixed frequency cutoff filters while light-adapted (LA) OPs are generally not isolated from ERGs. Our purpose was to analyze the effect of cutoff frequencies on DA and LA ERG components using a series of fixed and variable filters.

**Methods:**

DA and LA ERGs were recorded from 10 healthy eyes of 10 subjects (mean age = 20.5 ± 6.7 years) following ISCEV standards. Each signal was filtered in the Fourier domain to acquire slow (a- and b-waves; below cutoff frequency) and fast (OPs; above cutoff frequency) components. Fixed cutoff frequencies ranged from 60 to 105 Hz and a variable cutoff frequency was calculated. Results were analyzed with statistical tests and specific models.

**Results:**

DA ERG components were slightly influenced by the filter cutoff frequency. In contrast, fixed and variable filters significantly changed LA components: the lower the cutoff frequency the smaller the b-wave and OP3 and the higher the OP2/OP4 amplitudes. Analyzing the filter frequency limits a transition range between 68.9 Hz and 83.9 Hz was observed where amplitudes vary.

**Conclusions:**

The present report shows that DA OPs may be isolated from ERGs using filtering procedures with high-pass cutoff frequency at about 75 Hz as recommended by ISCEV. On the other hand, the spectral distribution of low-frequency and high-frequency LA ERG components may overlap. Accordingly, filtering the signal using different cutoff frequencies is not necessarily separating b-wave and OPs.

## Introduction

The electroretinogram (ERG) is a mass retinal bioelectric potential elicited by flashes of light with a combination of slow and fast components. Digital filters applied to ERG signals have been in use for a long time in an attempt to identify signal components, such as oscillatory potentials (OPs), that are hardly detected within the full signal spectrum usually recorded in the clinical routine [1–7]. Although, the cutoff frequencies to extract OPs from the original digitized dark-adapted (DA) ERG signals having a large range of frequencies up to 300 Hz is standardized by ISCEV, the influence of higher cutoff frequencies on OPs is still debated [8–10]. Moreover, light-adapted (LA) OPs are not necessarily extracted from the original digitized ERG signals during clinical examinations [11]. The fast OP components are usually observed when the slow components are reduced or eliminated by filtering out low-frequencies, for instance below 75 Hz, as recommended by the ISCEV, for the time domain analysis of DA OPs [11].

On the other hand, high-frequency components (OPs) are usually not filtered out from the original digitized ERG signals when the low-frequency components (a-wave and b-wave) are analyzed in the time domain. Although for most clinical cases this might not be relevant, OPs overlapping the slow components may influence the selection of a-wave and b-wave peaks in retinal conditions.

The purpose of this study was to evaluate the effects of different cutoff frequencies to DA and LA ERG components considering low and high frequency signal components. While OP changes associated with the cutoff frequency have been well described, the influence of the high-frequency components to the human a-wave and the b-wave is less known. We selected a range of frequencies (60 to 105 Hz) based on a previous report showing specific bands of temporal frequencies in the power spectrum of LA ERG signals [12]. In addition, a variable filter, as used in murine ERGs [13] has been applied.

### Methods

### Data collection

The experiment was performed in accordance with the declaration of Helsinki and approved by the institutional ethics committee. Participants were 10 healthy volunteers (mean age = 20.5 ± 6.7 years old; four males and six females) signing a consent to participate in the study after the explanation of the nature and possible consequences of the study. ERGs were recorded from one randomly selected eye using the RETiport system (Roland Consult, Brandenburg, Germany) equipped with Q450 SC Ganzfeld stimulator after i) eye dilation (tropicamide 1%), ii) electrodes placement: active DTL fiber electrode attached from the outer to the inner canthus, and ground / reference skin electrodes on the external canthi and forehead, respectively, and iii) a 20 min period of dark-adaptation. ERGs to dark-adapted (DA) and light-adapted (LA) 3.0 cd.s/m^2^ flash intensity (30 cd/m^2^ background luminance delivered for 10 min prior to LA recordings) were recorded according to ISCEV standards [11]. ERG signals with 512 points in 256 ms, including a 16 ms pre-stimulus, were obtained.

### Signal analysis

**Fig. 1:**
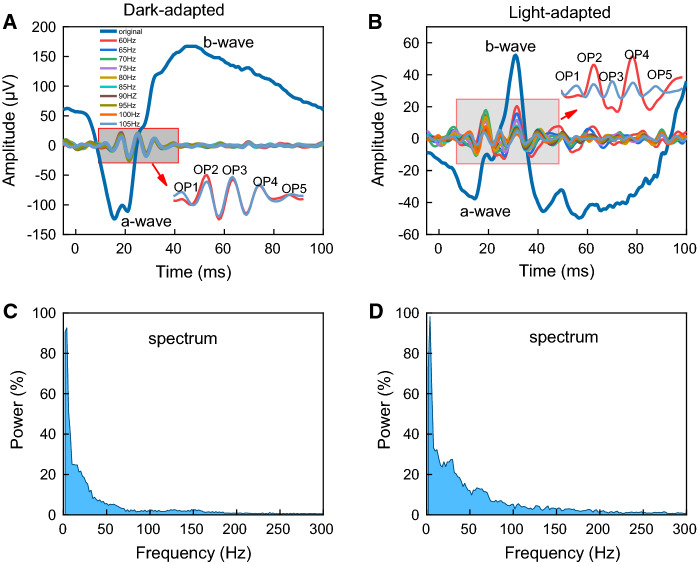
Averaged dark-adapted (**a**) and light-adapted (**b**) original ERG signals and OPs derived from the original ERG signals using a series of fixed high-pass cutoff filters from 60 to 105 Hz every 5 Hz. The name of the slow components (a-wave and b-wave) and the fast components (OP1 to OP5) analyzed are given. Averaged spectral power distributions are shown for the dark-adapted (**c**) and the light-adapted (D) ERG

Offline signal analysis was performed with self-written Matlab® (Mathworks, Natick, MA, USA) programs. Simple bandpass filters using Fast Fourier Transform (FFT) and Inverse Fast Fourier Transform (IFFT) were used. The spectral distribution with a bandwidth from 1 to 300 Hz in increments of 3.9 Hz, selecting only the frequency values to obtain approximately 5 Hz intervals from 60 to 105 Hz, were defined as cutoff frequencies for the fixed filter to separate the full range (1 to 300 Hz) into two frequency bands. Then the two new signals in the time domain: the low-frequency signal with slow components (a- and b-wave), and the high-frequency signal with fast components (OPs), were obtained with the IFFT. In addition, the spectral distribution of each individual signal was used to find the frequency with the first minimum amplitude between 60 and 90 Hz. This frequency was selected as the cutoff frequency for the application of the variable filter [13]. The original signals as well as the filtered signals were analyzed in the time-domain. The negative amplitudes (a-wave) were measured from the baseline (average amplitude of the first 5 ms recorded before the flash onset) to the trough while the amplitudes of the positive components (b-wave and positive OP peaks) were measured from the trough of the preceding negative peak. Peak times were measured from the flash onset to the peak amplitude of each component. Figure 1 shows average signals with components analyzed in the time domain (A: DA response and B: LA response) and the spectral distribution in the frequency domain obtained with the FFT (C: DA response and D: LA response).

### Statistical analysis

Data are expressed as means ± standard error of the mean. The effects of cutoff frequency filtering on ERG components were evaluated using Wilcoxon tests and paired t-test comparisons with Bonferroni post-hoc analyses (SPSS, Statistical Package for the Social Sciences, Hong Kong, China); multiple comparison corrected p values < 0.001 were considered statistically significant. In addition, Hill functions were fitted on measurement data, where applicable, to calculate the 50% thresholds and corresponding frequencies.

## Results

### Time-to-frequency and frequency-to-time domain analysis using fixed filters

Mean dark-adapted (DA; Fig. 1a) and light-adapted (LA; Fig. 1b) original ERG signals and OPs derived from the original ERG signals are shown in Fig. 1. Table 1 shows the mean (± standard error) of each component obtained with the original signals measured in the time domain. OPs were derived from the original ERG signals using a series of 10 fixed high-pass cutoff filters from 60 to 105 Hz. Individual time-domain amplitudes and peak times were calculated for each filter applied. Five oscillatory potentials were identified in both DA (Fig. 1a) and LA (Fig. 1b) signals. Peak time of the DA OP1 and OP2 coincided with a-wave peak time while DA OP3, OP4, and OP5 were superimposed to the rising slope of the signal which originates the b-wave. OP3 was usually the highest in amplitude (Fig. 1a, colored traces superimposed onto the original signal). LA OP1 was also superimposed onto the a-wave while OP2, OP3, and OP4 were superimposed onto the ascending limb of the b-wave. OP5 is after the b-wave. One may observe that LA OP amplitudes were largely and differently affected by the filtering condition (Fig. 1b), contrary to what was seen in DA OP responses (Fig. 1a). LA OP2 and OP4 amplitudes were about 49% and 52% lower at 105 Hz when compared to the amplitudes obtained at the 60 Hz cutoff frequency while LA OP3 amplitudes increased 54%, in average, at 105 Hz compared to the average at the 60 Hz cutoff frequency. LA OP3 amplitudes were larger than LA OP2 and OP4 amplitudes at 105 Hz cutoff frequency (Fig. 1b).

**Table 1:** DA and LA amplitudes and peak times (mean ± standard error) of the a-wave and the b-wave obtained from the original signals and after applying the variable filter

		Original		Variable filter	
		Amplitude (μV)	Peak time (ms)	Amplitude (μV)	Peak time (ms)
Dark-adapted	a-wave	197.6 ± 18.1	16.5 ± 0.7	196.2 ± 21.1	16.7 ± 0.4
	b-wave	305.9 ± 36.1	45.7 ± 2.6	298.7 ± 36.7	40.0 ± 1.8
Light-adapted	a-wave	42.4 ± 8	13.9 ± 0.6	38.3 ± 6.1	11.7 ± 0.6
	b-wave	114.2 ± 11.1	30.5 ± 0.4	98.4 ± 10.1	30.5 ± 0.3

Group averages of the spectral power distribution are shown in Fig. 1c (DA) and Fig. 1d (LA). DA and LA spectra differ in spectral power distribution especially between 60 and 105 Hz where the averaged LA power is more than twice as large (207%) as the average DA power.

### Time-to-frequency and frequency-to-time domain analysis using the variable filter

**Fig. 2:**
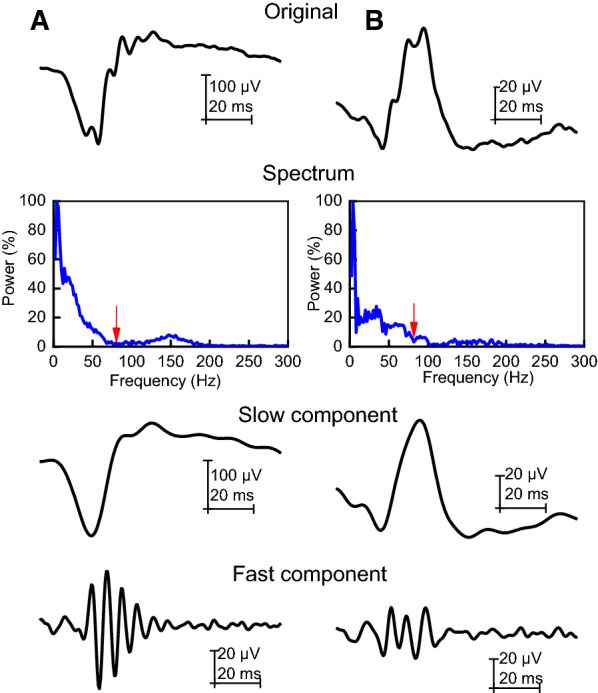
Variable filter applied to one representative subject for the DA (**a**) and LA (**b**) responses. The original ERG signals in the time domain are shown in the top row. Using the spectral power distribution (second row) the first minimum amplitude between 60 and 90 Hz was selected (indicated with the red arrow: 84 Hz and 82 Hz, respectively) as the cutoff frequency. The isolated low-frequency signal and high-frequency signal obtained from the original ERG signal using the frequencies below (slow component) and above (fast component) the cutoff frequency is shown in the third and fourth rows

In addition to the 10 fixed cutoff filters, a variable filter method has been applied to each individual signal. As previously described in the methods [13], the filter aimed to select the cutoff frequency as the first minimum amplitude of the spectrum calculated from each individual ERG signal. Figure 2 shows the analysis of one representative subject for the DA (Fig. 2a) and LA (Fig. 2b) responses. Similarly, to the analysis with the fixed cutoff filters, slow and fast components were derived from the original signals providing a-wave, b-wave, and OPs to be analyzed in the time domain. An individual spectral distribution is shown (Fig. 2) with red arrows indicating the minimum frequency between 60 and 90 Hz where the transition between slow and fast component separation generally occurs. The minimum FFT amplitude ranged from 72 to 85 Hz in both DA (mean = 78.2 ± 1.7 Hz) and LA (mean = 76.8 ± 1.4 Hz) conditions. Therefore, each individual signal showed a slightly different minimum frequency. Mean DA and LA slow component (a- and b-wave) amplitudes and peak time obtained from the variable filter are shown in Table 1 and were not statistically different from those of the values obtained with the original signal (p ≥ 0.02).

### Effects of the cutoff frequency on the slow (a-wave and b-wave) and fast (OPs) components of the DA ERG

**Fig. 3:**
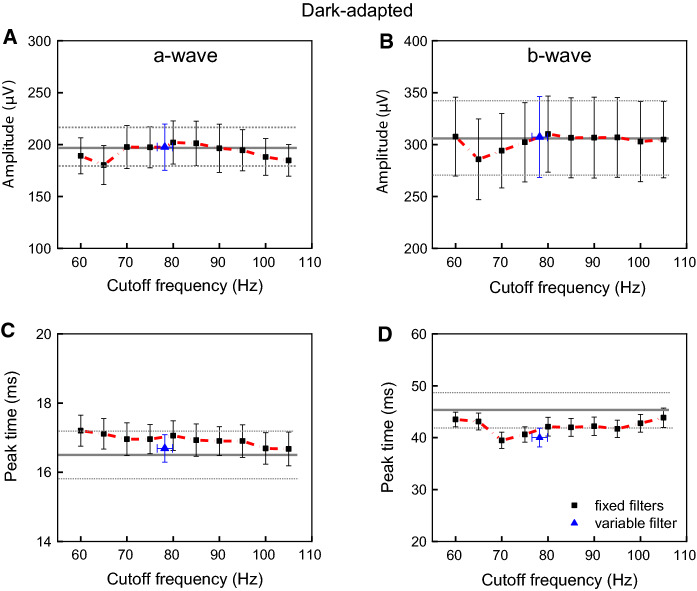
Dark-adapted slow components (a-wave and b-wave) response as a function of cutoff frequencies. Mean (± one standard error) amplitudes (**a**, **b**) and peak times (**c**, **d**) of the slow components (a-wave = A and b-wave = B) for each fixed cutoff frequency (connected by black symbols) and average (± standard error) variable filter (isolated blue symbol) are shown. Mean (± standard error) values obtained with the original signals are shown with horizontal gray lines and dotted lines

**Fig. 4:**
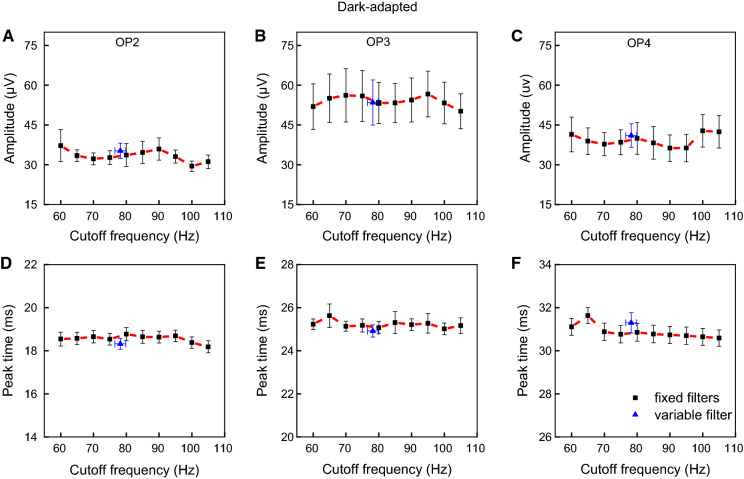
Dark-adapted fast components responses as a function of cutoff frequencies. Mean (± one standard error) amplitudes (upper plots) and peak times (lower plots) of the fast (**a** = OP2, **b** = OP3 and **c** = OP4) components for each cutoff frequency (connected black symbols) and average (± standard error) variable filter (isolated blue symbols) are shown

DA slow and fast components varied slightly in waveform among the 10 fixed filters. Figures 3 and 4 show mean (± standard error) amplitudes (upper plots) and peak times (lower plots) of the slow (a-wave and b-wave) and fast (OP2, OP3, OP4) components at each cutoff frequency set for the fixed filters (connected black symbols with short dash line), and the variable filter (isolated blue symbols). The mean (± standard error) values of the original a-wave and b-wave amplitudes and peak times are also displayed (horizontal gray lines and dotted lines).

The amplitudes of DA a- wave (Fig. 3a) and b-wave (Fig. 3b) did not significantly differ among the 10 filters applied and compared to the results obtained with the original signals (*p* > 0.02). In addition, the variable filter (mean = 78.2 ± 5.3 Hz) resulted in similar amplitude values (a-wave: *p* > 0.26; b-wave: *p* > 0.19) compared to the fixed filters. Peak times (Fig. 3c and d) were statistically similar among the 10 fixed cutoff filters (a-wave *p* > 0.14 and b-wave *p* > 0.98), although they were lower compared to the original signal for the b-wave, as shown in Fig. 3d.

Figures 4A to 4C show that DA OP2, OP3, OP4 amplitudes, respectively, obtained with ten fixed filters (OP2: *p* > 0.03; OP3: *p* > 0.09; OP4: *p* > 0.06) and the variable filter (OP2: *p* > 0.02; OP3: *p* > 0.16; OP4: *p* > 0.03), did not significantly change, considering correction for multiple comparisons (to reach statistical significance p-value should be < 0.001). Peak times were relatively constant for each fast component among fixed and variable filters (OP2: *p* > 0.07; OP3: *p* > 0.04; OP4: *p* > 0.11) as shown in Figs. 4d–f.

### Effects of the filters on the slow (a-wave and b-wave) and fast (OPs) components of the LA ERG

**Fig. 5:**
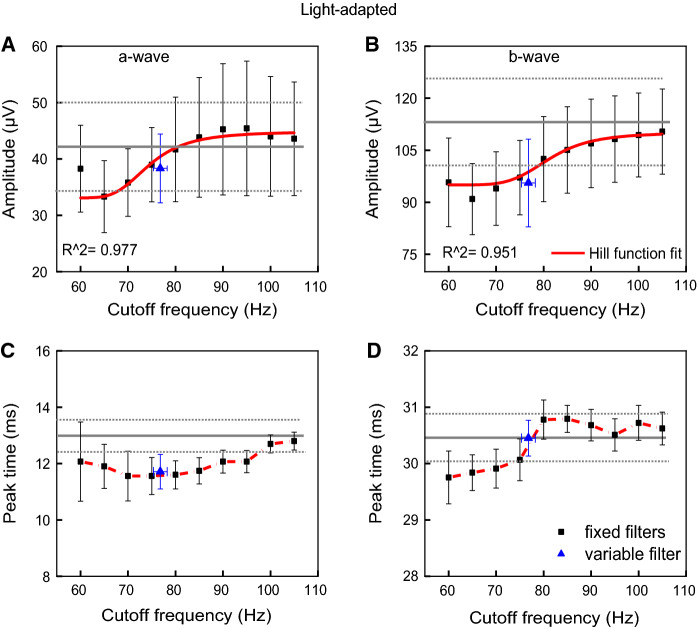
Light-adapted (LA) slow components (a-wave and b-wave) as a function of cutoff frequencies. Mean (± standard error) amplitudes (**a** and **b**) and peak times (**c** and **d**) for each cutoff frequency (connected black symbols) and the average (± standard error) variable filter (isolated blue symbols) were shown. Mean (± standard error) values obtained with the original signals are shown with horizontal gray lines and dotted lines. Hill function was applied to the a- and b-wave amplitudes (**a** and **b**) showing that amplitudes increase as a function of cutoff frequency achieving a level at about 85 Hz with the 50% threshold at (76.2 ± 8.9) Hz and 79.4 (± 4.5) Hz, respectively

**Fig. 6:**
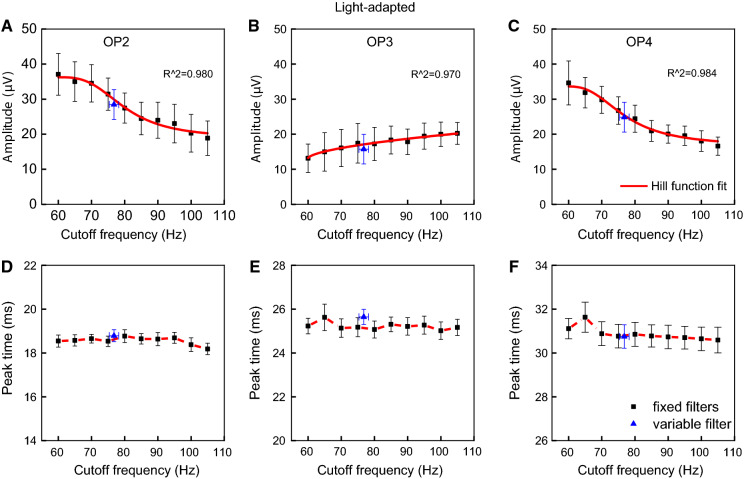
Light-adapted (LA) fast components (OP2, OP3, and OP4) as a function of cutoff frequencies. Mean (± one standard error) amplitudes was fitted with the Hill function (upper row) and peak times (lower row) at each cutoff frequency set for the fixed filters (connected black symbols) and average (± standard error) variable filter (isolated blue symbols) are shown. A transition range of the fast components is between 68.9 and 83.9 Hz with a frequency of 75.9 (± 3.5) Hz at 50% threshold of the Hill function

Contrary to the effects on DA ERG, LA slow and fast components significantly changed with the filters. Figures 5 and 6 show the mean (± standard error) amplitudes (upper plots) and peak times (lower plots) of the LA slow (a-wave and b-wave) and the fast (OPs) components for the fixed filters with different cutoff frequencies (connected black symbols), and for the variable filter (isolated blue symbols). The mean (± standard error) values of the original a-wave and b-wave amplitudes and peak times are also displayed (horizontal gray lines and dotted lines).

Hill equation was applied to analyze LA amplitudes of all filtered components. The fitting curves show “S-like” shapes, which means there’s a linear range corresponding to the middle section (transition range) and two relatively constants sections at low and high frequencies. LA a-wave amplitudes varied among the fixed filters with the 50% threshold at 76.23 (± 8.90) Hz, but not significantly (*p* = 0.16, comparing the lowest amplitude at 65 Hz with the highest amplitude at 105 Hz), as shown in Fig. 5a. In addition, a-wave amplitudes obtained with the fixed filters did not differ statistically from the original a-waves (*p* > 0.02), considering correction for multiple comparisons (to reach statistical significance p-value should be < 0.001). The amplitude of b-wave is shown in Fig. 5b. The results transited from a relatively constant low sections to a high section with the 50% threshold at 79.4 (± 4.5) Hz, in average. The b-wave amplitudes obtained with cutoff frequencies higher than 80 Hz were not significantly different from the original signal’s b-wave (*p* > 0.30). Moreover, amplitudes obtained with the fixed filters and the variable filter (76.8 ± 1.4 Hz) did not differ significantly (a-wave: *p* > 0.07; b-wave: *p* > 0.02). On the other hand, amplitudes obtained with low and high cutoff frequencies (60 Hz vs. 80-105 Hz *p* < 0.0007; 65 Hz vs. 95-105 Hz *p* < 0.0004; 70 Hz vs. 100-105 Hz *p* < 0.0001; 80 Hz vs. 95-105 Hz *p* < 0.0006) differed significantly from each other, indicating significant effect of the filter frequency on LA b-wave amplitudes. Peak times of the slow components (Figs. 5c and d) varied among the 10 fixed cutoff filters (a-wave: 95 Hz vs. 100–105 Hz p < 0.0004, and b-wave: 65 Hz vs. 80–95 Hz *p* < 0.0006) indicating significant effect of the filter frequency on LA slow components. Moreover, the peak times of both a-wave and b-wave obtained with the fixed filters and the variable filter (76.8 ± 1.4 Hz) did not differ significantly (a-wave: *p* > 0.09; b-wave: *p* > 0.001 0.99) Statistical values showed comparable a-wave peak times, although in Fig. 5c the mean value obtained with the variable filter was below the low standard error value of the mean a-wave peak time obtained with original signals. However, we assume that this slight, non-significant, change is observed because the negative component of the original signal is influenced by initial OPs overlapping the a-wave.

The amplitudes of OP2 (Fig. 6a) and OP4 (Fig. 6c) decreased with increasing cutoff frequency of the filter. The 50% threshold was 81.4 ± 6.1 Hz and 75.5 ± 5.1 Hz, respectively, while the amplitude of OP3 increased with increasing cutoff frequency of the filter with the 50% threshold at 75.4 ± 6.1 Hz. OP2 and OP4 showed higher amplitudes at low cutoff frequencies and lower amplitudes and high cutoff frequencies with significant differences even after applying multiple comparison’s correction (*p* < 0.001) to the pairwise comparisons (OP2: 60-65 Hz vs. 95-100 Hz, 70-75 Hz vs. 90-105 Hz, 80 Hz vs. 105 Hz; OP4: 65-70 Hz vs. 75-105 Hz). Calculating the frequency limits of all transition zones, a range between 68.9 and 83.9 Hz with a frequency of 75.9 ± 3.5 Hz at 50% threshold was found. Peak times of the fast components were relatively constant among the 10 fixed cutoff filters and the variable filter (OP2: *p* > 0.52; OP3: *p* > 0.03; OP4: *p* > 0.49), as shown in Fig. 6d–e.

## Discussion

**Fig. 7:**
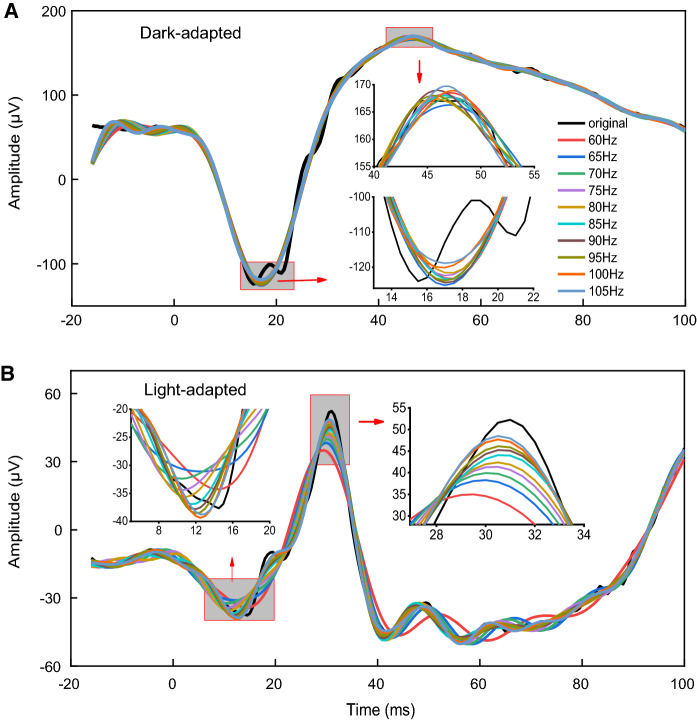
Slow components (a-wave and b-wave) derived from the original ERG signals (grey traces) using 10 fixed cutoff frequencies (colored traces). Amplified plots for the peak responses are shown. Isolated DA a-wave and b-wave are relatively stable when OPs are excluded from the original signals and they resemble those obtained with the original signal (**a**). LA a-wave and b-wave changed significantly with OPs excluded from the signal (**b**). The changes depended on the cutoff frequency: as higher the cutoff frequency higher the b-wave amplitude and peak time (more delayed response)

Dark-adapted (DA; scotopic) oscillatory potentials (OPs) showed comparable amplitudes and peak times when they were extracted from the original ERG signals using post hoc filtering at frequencies between 60 and 105 Hz. Moreover, the amplitudes of isolated DA a-wave and b-wave were relatively stable when OPs were removed from the original signals using cutoff frequencies between 75 and 105 Hz (Fig. 7a). In addition, DA low-frequency components (a-wave and b-wave) obtained with the variable filter resembled those obtained with cutoff filters at this frequency interval (75 to 105 Hz) and the original signal. In contrast, significant changes were found in light-adapted (LA; photopic) ERG, b-wave (Fig. 5b) and OP (Fig. 6a–c) amplitudes depending on the filter cutoff frequency. A transition of the b-wave amplitude was observed from relatively stable low values to high values in a frequency range with 50% threshold at 79.4 (± 4.5) Hz, in average. LA b-wave was affected by all cutoff frequencies, as expected from a previous report [14], because of OP4 and b-wave coincident peak times. In addition, the application of the variable filter based on the minimum amplitude of the LA temporal frequency spectrum (see Fig. 2) resulted in amplitudes and peak times similar to the ones obtained with 50% threshold of the fitted Hill function at 79.4 (± 4.5) Hz. In summary, the application of the variable filter that effectively separates low- and high-frequency components in mice [13, 15, 16] does not seem to be necessary for analyzing human DA and LA ERGs. We recognize that flash strength of 3 cd.s/m^2^ does not provide well-defined LA OPs [17], however this is the ISCEV standard flash strength widely used in clinical examinations.

LA OP2 and OP4 amplitudes decreased as the cutoff frequency increased while LA OP3 amplitudes increased with increasing the cutoff frequency. Therefore, high-pass filters with cutoff frequencies set at lower values (separating high and low frequency components below 68.9 Hz according to present data) may emphasize OP2 and OP4, while in the lower frequency range it may generate a more isolated b-wave, less contaminated by the OP frequencies. In contrast, higher cutoff frequencies (90–105 Hz) beyond the transition range, result in larger b-wave and lower OP amplitudes, most likely due to b-wave-OP4 interactions (coinciding peaks). The present results, therefore, confirm previous investigations showing that specific cutoff frequencies may influence the main ERG components, particularly the photopic b-wave (Fig. 7b), as well as the extraction of photopic OPs [18]. These findings highlight the need for further clinical investigations concerning the influence of filtering frequencies to isolate slow and fast components of the ERG in different retinal conditions.

OPs are elicited at the same time or very closely synchronized with the slow ERG components [19, 20]. Frequency spectrum analysis and filtering methods to derive human OPs from the original ERG signals have been extensively investigated in the past decades [3–5, 10, 12, 21–25]. It has been reported that the main components of the DA ERG influence OP amplitudes, as the slow ERG components (a-wave and b-wave), usually measured with a broad-spectrum band up to 300 Hz, can also be contaminated with high frequency components. Following ISCEV standard recommendations [11], digital filters are usually integrated to the software in commercially available ERG systems with fixed cutoff frequencies, generally between 75 and 100 Hz, to record isolated OPs in the DA ERG protocol. The present results show that indeed at this frequency range DA OP amplitudes and peak times are nearly indistinguishable independent of the specific frequency used. Moreover, if OPs are removed from the original DA signal allowing the isolated analysis of slow components (a-wave and b-wave) using cutoff frequencies between 75 and 100 Hz, the original amplitudes and peak times of the a-wave and the b-wave may only change slightly (Fig. 7a). Therefore, cutoff frequencies between 75 and 100 Hz, as recommended by ISCEV, may be used to derive DA OPs from ERG signals as long as the same filter is used in a single clinical or research center allowing reliable comparisons among subjects. The present results showed that in humans the application of the variable filter provides results similar to the ones obtained with the original signal (without excluding the OPs) for the main ERG components (a-wave and b-wave). Moreover, DA OP amplitudes and peak times using the variable filter (mean = 78.2 Hz) resembled those obtained with the ISCEV standard 75 Hz filter.

LA OPs differ from DA OPs in terms of amplitude, peak time, and frequency, since LA ERG spectrum has two main bands of high temporal frequencies, one between 70 and 80 Hz and the other between 130 and 160 Hz [9, 26–28]. The characterization of LA OPs with bandpass filtering has been recently reported by Gauthier et al. [12]. Previously, the same group reported a series of experiments using different types of bandpass filters combined with variable bandwidth ranges and, in addition to FFT, continuous wavelet transform (CWT), and discrete wavelet transform (DWT) were applied for a detailed characterization of the LA signals. In summary, Gauvin.et al.’s investigations demonstrated that the frequency distribution of the LA OPs is bimodal and spectral components manipulation may considerably influence LA ERG responses in pathological conditions [27–29]. As isolated LA OPs are usually not considered in the clinical context they may be useful in identifying early pathological changes in the retina [28, 30].

In the LA condition, slow (a-wave and b-wave) and fast (OPs) human ERG components are dependent on each other supporting the view that LA b-wave is shaped by the OPs. This, in turn, is supported by the findings that LA OP (summed) amplitude is proportional to the b-wave amplitude in normal eyes [18]. It has been long proposed that slow and fast ERG components do not share the same cellular origin since a-wave and b-wave mainly reflect photoreceptoral and bipolar cell activation, respectively [31, 32], while the generation of the OPs is attributed to inner retinal structures upstream the ganglion cells [33], most likely generated by neural feedbacks responsible for inhibitory synaptic transmissions [34, 35]. Accordingly, altered b-waves with normal OPs and vice-versa are often reported. For instance, slightly reduced to completely abolished OPs without a-wave and / or b-wave alterations have been found before any detectable clinical sign of diabetic retinopathy (DR). Moreover, LA OP changes may predict the progression of DR [36–38] and can be useful to evaluate toxic effects of pharmacological treatments and the subsequent retinal recovery following treatment discontinuation [39, 40]. Although amacrine cells are generally well-accepted as the OP generators [41, 42] with possible contribution from the bipolar cells [43], OPs may be sensitive to microvascular alterations of the retina [5]. The specific retinal origins of the OPs is also supported by the findings that LA OP2 and OP3 are absent in congenital stationary night blindness, while OP4 is spared [44]. Individual OPs may indeed be generated by specific structures of the retina [42], since pharmacological interventions blocking the activation of specific cells affect differently the OPs [45]. A selective origin of individual OPs emphasizes their clinical value [46], in addition to the clinical relevance of the main ERG components. The present data show that LA b-wave and OPs are differently affected by the cutoff frequency of the digital filters applied to original ERG signals in healthy retinas. Moreover, we found that cutoff frequencies above 100 Hz would result in LA a- and b-wave amplitudes remarkably similar to the amplitudes of the original signal, meaning that at this frequency the electrophysiologist could be safe to isolate LA OPs that should not be contaminated by the components of the b-wave. However, one needs to consider that the filtered frequencies (i.e.: below 100 Hz) also influencing OP amplitudes (mainly LA OP2 and OP4), are eliminated.

The study aimed to describe the effects of cutoff frequencies and the variable filter on low-frequency and high-frequency components. Further investigations of LA ERG signals using more complex time–frequency analysis and evaluating conditions affecting specific retinal structures may provide additional information regarding the application of specific cutoff frequencies in human LA ERGs.

## Conclusion

The present study investigated the effects of fixed cutoff frequency filtering on dark-adapted and light-adapted ERG components and the application of a variable filter showing that LA b-wave and OPs may have different amplitudes and peak times depending on the cutoff frequency in healthy ERG signals. Data obtained with the variable filter are comparable to those obtained with fixed filters. Further investigations may consider applying this analysis to ERG signals recorded from eyes with retinal diseases affecting the inner retina such as those specifically affecting LA OPs. It may also be worthy to investigate it in retinal conditions affecting photoreceptors and / or bipolar cells to evaluate the isolated (OP-free) LA b-wave.
